# Two hybrid flow shop scheduling lines with assembly stage and compatibility constraints

**DOI:** 10.1371/journal.pone.0304119

**Published:** 2024-06-21

**Authors:** Rafael Muñoz-Sánchez, Iris Martínez-Salazar, José Luis González-Velarde, Yasmín Á. Ríos Solís

**Affiliations:** 1 Facultad de Ingeniería Mecánica y Eléctrica, Universidad Autónoma de Nuevo León, San Nicolás de los Garza, Nuevo León, México; 2 School of Engineering and Science, Tecnológico de Monterrey, Monterrey, Nuevo León, México; Cyprus International University Faculty of Engineering: Uluslararasi Kibris Universitesi Muhendislik Fakultesi, TURKEY

## Abstract

Two hybrid flow shop scheduling lines must be coordinated to assemble batches of terminated products at their last stage. Each product is thus composed of two jobs, each produced in one of the lines. The set of jobs is to be processed in a series of stages to minimize the makespan of the scheduling, but jobs forming a product must arrive at the assembly line simultaneously. We propose a mixed integer linear programming model. Then, based on the model, we propose a pull-matheuristic algorithm. Finally, we present two metaheuristics, a greedy randomized adaptive search procedure and a biased random key genetic algorithm, and compare all the methodologies with real-based instances of a production scheduling problem in the automobile manufacturing industry. The greedy algorithm yields high-quality solutions, while the genetic one offers the best computational times.

## 1 Introduction

The scheduling of flow shops with multiple parallel machines per stage, referred to in the literature as the hybrid flow shop (HFS), flexible flow shop, or multi-processor flow shop [1–3], is a complex combinatorial problem encountered in many real-world manufacturing processes.

**Fig 1:**
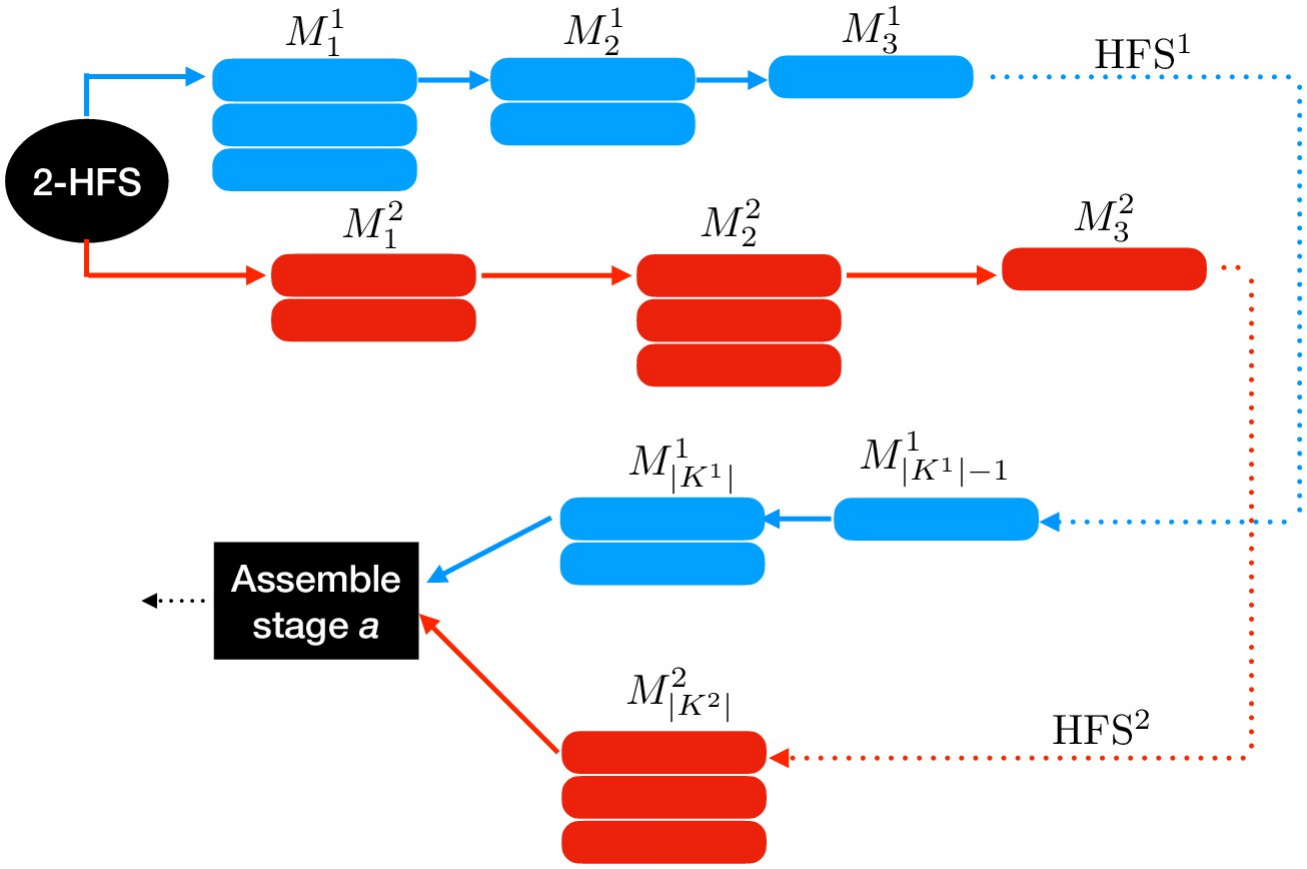
2-HSFA: Two HFS must be coordinated to assemble batches of terminated products at the end of the lines.

In this article, two HFS must be coordinated to assemble batches of terminated products at the end of the lines, as shown in Fig 1. Each product is thus composed of two jobs, each produced in one of the HFS. In both HFS, the set of jobs are to be processed in a series of stages to minimize the time that elapses from the start of jobs to their end, that is, to minimize the maximal completion time or *makespan* of the scheduling. The jobs forming a product must arrive at the assembly line simultaneously, even if one HFS is, on average, faster than the other. Each stage of the HFS lines has several unrelated parallel machines. In each HFS, jobs pass through the stages of the shop floor in a flow sequence, but a job might skip some stages if they are not necessary for its manufacture. Each job *j* requires a processing time *p*_*jk*_ in stage *k*. We name this problem the 2-Hybrid Flow Shop with Assemble stage (2-HFSA).

As is usual in HFS [4], we suppose that all jobs and machines are available at time zero. Machines can process only one job operation at a time, and job operations can be processed by only one machine at a time. Moreover, operation preemption is not allowed.

In [5], the shop problems are classified according to their configuration, constraints and assumptions, and objective function. In our case, each HFS denoted as HFS^1^ and HFS^2^, can be described as $FHm^{l},{\left(RM^{\left(k\right)}\right)}_{k=1}^{m^{l}}|prmu,M_{j},S_{sd}|C_{\text{max}}$, for *l* = {1, 2}. *FHm*^*l*^ indicates a hybrid flow shop with *m*^*l*^ stages. ${\left(RM^{\left(k\right)}\right)}_{k=1}^{m^{l}}$ means that in all stages, *k* = 1, …, *m*^*l*^, there are any number of unrelated parallel machines. The term *prmu* indicates that the jobs are processed in every stage in the same order. The processing of job *j* is restricted to the set of machines *M*_*j*_ at stage *k*, known as eligibility. *S*_*sd*_ indicates that the setup times depend on the sequence of operations. Finally, *C*_max_ is the maximum completion time.

Solving a single of our HFS problems is NP-hard. Indeed, our problem can be reduced to an HFS with two stages, even when one stage contains two machines and the other one a single machine, which is already NP-hard [6]. Notice that even if the objective functions of HFS^1^ and HFS^2^ correspond to the minimization of their makespan, a solution to the 2-HFSA problem may have long idle times because of the assembly constraint at the end of the production lines. This is where the main challenge of the 2-HFSA problem resides, in the coordination at the assembly stage that generates idle times.

This research’s case study takes place in the automobile parts production system, more precisely, in a tractor-truck parts manufacturer. Their main products are tractor axles, and their essential elements are the crown-pinion assembly. The crown-pinion line’s production times are significant and are the company’s bottleneck since it is the hardest to optimize. The HFS1 makes the crowns, while the HFS2 produces the pinions. These two pieces form a product. Since the manufacturing process continues with the crown-pinion product, the batches of these two pieces should arrive simultaneously at the assembly stage.

This work focuses on solving the 2-HFSA, which has an assembly stage of two HFS. First, we propose a Mixed Integer Linear Programming model (MILP) to assert the exact assembly time at which both parts should be assembled, together with the machine eligibility and sequence-dependent setup times. The problem is NP-hard, so the size of the instances that can be solved optimally is relatively small. Then, we propose a matheuristic algorithm that takes advantage of our mathematical model. It first solves the sequence, and then the timing is determined. Still, we propose two metaheuristics to face larger instances: a Greedy Randomized Adaptive Search Procedure (GRASP) and a Biased Random Key Genetic Algorithm (BRKGA). An extensive calibration of the different parameters and operators using experimental designs was executed. To evaluate the proposed algorithms, we conducted several experiments with 20 random instances based on real data from the case study company.

The rest of the paper is organized as follows. A literature review is presented in Section 1. The problem description and its mathematical model are in Section 2.1. Section 2.2 describes the matheuristic, while the metaheuristics algorithm GRASP and BRKGA are in Sections 2.3 and 2.4. Section 3 includes the experimental results, and Section 4 concludes the work.

### 1.1 Literature review

The HFS literature is large, but excellent reviews show the main tendencies in the field [1, 7, 8]. For the $FHm,{\left(RM^{\left(k\right)}\right)}_{k=1}^{m}||C_{\text{max}}$ problem, [9] propose branch-and-bound algorithms. Also, [10, 11] determine lower bounds, while heuristics and metaheuristics are proposed in [12–15]. Moreover, neural networks are presented in [16], and ant-colony algorithms in [17]. The authors include sequence-depending setup times in [18–21], propose mathematical formulations and metaheuristics. For the $FHm,{\left(RM^{\left(k\right)}\right)}_{k=1}^{m}|M_{j}|C_{\text{max}}$ problem, which is more related to ours because of the restricted machines per stage, [22, 23] propose genetic algorithms. Nevertheless, authors do not consider an assembly stage of the coordination of two lines.

Some applications require more specific characteristics. [24] deal with $FHm_{l},{\left(RM^{\left(k\right)}\right)}_{k=1}^{m}|reentry,batch,S_{sd}|C_{\text{max}}$ problem in a cardboard company where the system required re-entrant flows, external operations, and transfer batches between stations. [25] solve the $FHm_{l},{\left(RM^{\left(k\right)}\right)}_{k=1}^{m}|M_{j},S_{sd},buffer|C_{\text{max}}$ problem with a genetic algorithm that takes into account the sequence-dependent setup time, availability constraints, and limited buffers. [26] solve the $FHm_{l},{\left(RM^{\left(k\right)}\right)}_{k=1}^{m}|M_{j},S_{sd}|C_{\text{max}}$ problem in the textile industry and the synthetic paint business with mathematical programming and genetic algorithms. In [27], the authors study an agricultural product scheduling problem. They introduces a mixed integer linear programming mathematical model to minimize work in process and maximize average machine cell utilization. However, none of these works considers the coordination of two different lines as the one proposed in this research.

[28] were among the first to consider assembly lines. They solve the $FH2,({\left(2^{\left(1\right)},P2^{\left(2\right)}\right)}_{k=1}^{m}|assembly^{\left(2\right)}|\bar{F}$ problem with only two stages and only one of two machines per stage. The processing of the second stage cannot begin until the processing of the first stage is finished. The authors propose a lower bound, a branch-and-bound algorithm, and a simple heuristic algorithm. [29] study an HFS with assembly operations. The parts are produced in a hybrid flow shop, and an assembly stage produces the final products. A hierarchical branch-and-bound algorithm is presented. [30] determine the assembly scheduling and transportation allocation to minimize the waiting times. The main difference with our problem is that [30] consider only one HFS.

[31] present a two-stage HFS problem followed by a single assembly machine as the one we study in this article. As in our case, parts must be processed on the HFS stages and joined in the assembly stage to produce the final product. The authors propose two metaheuristic techniques. [32] propose a hybrid solving method that combines improved extended shifting bottleneck procedure and genetic algorithm for the assembly job shop scheduling problem, which differs from the one we propose here. [33] analyze a production line in an automobile assembly plant, using simulation and dispatching rules, to define a production planning strategy for the company. [34] focus on the green scheduling problem in a flexible job shop system. The authors formulate a mixed integer linear multiobjective optimization model. Recently, [35] tackle the hybrid flow shop scheduling problem with a heterogeneous graph neural network to learn an optimal scheduling policy.

## 2 Materials and methods

### 2.1 Mixed integer linear programming for the 2-HFSA problem

Let set *J*^*l*^ be the set of jobs manufactured by the HFS^l^, and *J* = *J*^1^ ∪ *J*^2^ be the set of jobs of the 2-HFSA problem. Let *N* = {(*i*, *j*)∣*i* ∈ *J*^1^, *j* ∈ *J*^2^} be the set of final products to be assembled. The stages of the 2-HFSA problem are the set *K* = *K*^1^ ∪ *K*^2^ ∪ *a* where *a* corresponds to the assembly stage (see Fig 1). Similarly, we define the machine set as $M=\cup_{l\in L,k\in K^{l}}M_{k}^{l}$.

The processing time of job *j* ∈ *J*^*l*^ in stage *k* ∈ *K*^*l*^ on machine $m\in M_{k}^{l}$ is $p_{jkm}^{l}$. Note that every job’s processing time in the assembly stage *a* is null. Thus, $p_{ja}^{l}=0$, for *j* ∈ *J*. Jobs pass through the stages of the shop floor in a flow sequence, but a job might skip some stages if they are not necessary for its manufacture. Thus, eligibility is represented by the parameter $e_{jkm}^{l}$, which takes a value of one when job *j* ∈ *J*^*l*^ must be executed with machine $m\in M_{k}^{l}$ of stage *k* ∈ *K*^*l*^ for component *l* ∈ *L*. Moreover, let $\tau_{(i,j)km}^{l}$ represent the setup time between the job pair (*i*, *j*) in machine $m\in M_{k}^{l}$, for *i*, *j* ∈ *J*^*l*^ and component *l* ∈ *L*.

To model the 2-HFSA problem with a mixed integer linear programming formulation, let binary decision variables $y_{jkm}^{l}$ take a value of one if job *j* ∈ *J*^*l*^ is assigned to machine $m\in M_{k}^{l}$ in stage *k* ∈ *K*^*l*^, for component *l* ∈ *L*, and zero, otherwise. To determine the sequence of the jobs, let binary variables $x_{(i,j)km}^{l}$ take a value of one if job *i* ∈ *J*^*l*^ is scheduled immediately before job *j* ∈ *J*^*l*^ in machine $m\in M_{k}^{l}$, at stage *k* ∈ *K*^*l*^, component *l* ∈ *L*, and zero, otherwise. Auxiliary variables $C_{jk}^{l}$ represent the completion time of job *j* ∈ *J*^*l*^ in stage *k* ∈ *K*^*l*^ ∪ *a* for component *l* ∈ *L*. Similarly, $S_{(i,j)km}^{l}$ denotes the starting time of job *j* ∈ *J*^*l*^, scheduled immediately after job *i* in stage *k* ∈ *K*^*l*^ in machine $m\in M_{k}^{l}$, and component *l* ∈ *L*. Finally, *C*_*max*_ represents the *makespan* of the 2-HFSA problem. The mathematical model for the 2-HFSA is as follows.

$$\begin{array}{c} \text{min}\;C_{\text{max}} \end{array}$$ (1)

$$\begin{array}{ccc} & C_{ja}^{l}\leq C_{\text{max}} & j\in J^{l},\;l\in L \end{array}$$ (2)

$$\begin{array}{ccc} & y_{jkm}^{l}\leq e_{jkm}^{l} & i\in J^{l},k\in K^{l},m\in M_{k}^{l},l\in L \end{array}$$ (3)

$$\begin{array}{ccc} & \sum_{m\in M_{k}^{l}}e_{jkm}^{l}y_{jkm}^{l}=1 & k\in K^{l},j\in J^{l},l\in L \end{array}$$ (4)

$$\begin{array}{ccc} & x_{(i,j)km}^{l}-x_{(j,i)km}^{l}\leq 1 & i,j\in J^{l},k\in K^{l},m\in M_{k}^{l},l\in L \end{array}$$ (5)

$$\begin{array}{ccc} & x_{(i,j)km}^{l}\leq y_{jkm}^{l} & i,j\in J^{l},k\in K^{l},m\in M_{k}^{l},l\in L \end{array}$$ (6)

$$\begin{array}{ccc} & \sum_{j\in J^{l}}e_{jkm}^{l}x_{(0,j)km}^{l}\leq 1 & k\in K^{l},m\in M_{k}^{l},l\in L \end{array}$$ (7)

$$\begin{array}{ccc} & \sum_{i\in J^{l}\cup 0}x_{(i,h)km}^{l}-\sum_{j\in J^{l}\cup 0}x_{(h,j)km}^{l}=0 & h\in J^{l},m\in M_{k}^{l},k\in K^{l},\;l\in L \end{array}$$ (8)

$$\begin{array}{ccc} & \sum_{j\in J^{l}}e_{jkm}^{l}x_{(j,0)km}^{l}\leq 1 & m\in M_{k}^{l},k\in K^{l},l\in L \end{array}$$ (9)

$$\begin{array}{c} C_{jk}^{l}\geq S_{(i,j)km}^{l}+p_{jkm}^{l}y_{jkm}^{l}+\tau_{(i,j)km}^{l}x_{(i,j)km}^{l}-B\left(1-x_{(i,j)km}^{l}\right)\\ i,j\in J^{l},k\in K^{l},m\in M_{k}^{l},l\in L \end{array}$$ (10)

$$\begin{array}{ccc} & S_{(i,j)km}^{l}\geq C_{jk-1}^{l} & i,j\in J^{l},k\in K^{l},k>1,l\in L \end{array}$$ (11)

$$\begin{array}{cc} C_{ia}^{1}-C_{ja}^{2}=0 & (i,j)\in N \end{array}$$ (12)

$$\begin{array}{ccc} & x_{(i,j)km}^{l},y_{ikm}^{l}\in \left\{0,1\right\} & i,j\in J^{l},m\in M_{k}^{l},k\in K^{l},l\in L\\ & C_{jk}^{l},S_{(i,j)km}^{l},C_{\text{max}}\geq 0 & i,j\in J^{l},m\in M_{k}^{l},k\in K^{l},l\in L \end{array}$$

Expression (1) represents the makespan objective function. Constraints (2) define the makespan as the completion time of the last job processed in the assembly stage *a*. Expressions (3) assign a job *j* ∈ *J*^*l*^ to a machine $m\in M_{k}^{l}$ in stage *k* ∈ *K*^*l*^ only if the machine is eligible, *l* ∈ *L*. Eq (4) imply that each job *j* ∈ *J*^*l*^ must be assigned to a machine $m\in M_{k}^{l}$ in every stage *k* ∈ *K*^*l*^, for *l* ∈ *L*. Constraints (5) prevent cycling between two consecutive jobs and define a unique sequence. Constraints (6) state that a job *j* ∈ *J*^*l*^ cannot be sequenced in machine $m\in M_{k}^{l}$ in stage *k* ∈ *K*^*l*^ if not assigned to that machine. Constraints (7)–(9) ensure flow conservation in each machine by considering a dummy job 0 at the beginning and the end of the sequence. Constraints (10)) follow the idea proposed by [36] for sub-tours elimination purposes and establish the completion time of the jobs by considering the setup and the processing times; *B* is a big number that can be easily bounded. Constraints (12) indicate the simultaneous arrival of a product’s components at the assembly stage. Finally, the last two constraints establish the nature of the variables.

This mixed integer linear programming can solve small instances, as shown in Section 3. In the following, we propose a matheuristic, a GRASP, and a BRKGA to solve larger instances.

### 2.2 Matheuristic for the 2-HFSA problem

Matheuristics are hybrid methods that combine exact approaches with metaheuristic strategies that aim to acquire the accuracy of mathematical programming and the benefits of computational time consumption that heuristics offer. According to [37], matheuristics often exhibit a “master-slave” scheme, where one of the elements (exact or approximated approach) takes the master’s place and controls the other element. In the 2-HFSA problem, the job assignment to the machine stages corresponds to the master problem, while the scheduling of the jobs in the different machines is the slave problem. Both sub-problems are solved with a MILP but linked with a pull-planning heuristic method.

A pull system is a scheduling technique that fixes the project makespan of the jobs and then works backward to outline the steps to achieve the planned makespan quickly and efficiently [38]. In this manner, in our pull-planning matheuristic we aim for the simultaneous arrival of both product components at the assembly stage by finding feasible complying schedulings with our assignment subproblem. Unfeasible assignments are stored on a forbidden list to avoid cycling.

**Algorithm 1** Pull-matheuristic

1: Forbidden assignment solutions Γ = ∅

2: ${\bar{C}}_{\text{max}}=|J|({\text{max}}_{j\in J^{l},k\in K^{l},m\in M_{k}^{l},l\in L}p_{jkm}^{l}+{\text{max}}_{j\in J^{l},k\in K^{l},m\in M_{k}^{l},l\in L}\tau_{jkm}^{l})$

3: **while** maximum iterations without improvement not reached **do**

4:  solve AS(Γ) to obtain a non forbidden assignment $\bar{Y}$

5:  solve $SS(\bar{Y},{\bar{C}}_{\text{max}})$ to obtain a job scheduling *γ*

6:  **while** time limit is not reached **do**

7:   **if** $SS(\bar{Y},{\bar{C}}_{\text{max}})$ is feasible **then**

8:    **if** $SS(\bar{Y},{\bar{C}}_{\text{max}})$ objective function value > 0 **then**

9:     Γ ← Γ ∪ *γ*, ${\bar{C}}_{\text{max}}\leftarrow \left(1+\beta \right){\bar{C}}_{\text{max}}$

10:    **else** ${\bar{C}}_{\text{max}}\leftarrow \left(1-\beta^{\prime }\right){\bar{C}}_{\text{max}}$, update $Y^{*},C_{\text{max}}^{*}$

11:    **end if**

12:   **else if**

13:    **then** ${\bar{C}}_{\text{max}}\leftarrow \left(1+\beta \right){\bar{C}}_{\text{max}}$

14:   **end if**

15:  **end while**

16: **end while**

Algorithm 1 presents the pull-planning matheuristic pseudocode. First, the forbidden assignment set Γ is empty, and the trivial bound ${\bar{C}}_{\text{max}}$ on the optimal makespan is set. While a maximum number of iterations without improvement has not been reached, the assignment subproblem AS(Γ) is solved, yielding a job-stage-machine unforbidden assignment $\bar{Y}$. This assignment is a parameter when solving the $SS(\bar{Y},{\bar{C}}_{\text{max}})$ model as well as ${\bar{C}}_{\text{max}}$. While the $SS(\bar{Y},{\bar{C}}_{\text{max}})$ model yields a positive solution (step 8), the algorithm increases the ${\bar{C}}_{\text{max}}$ by a percentage of *β* and the algorithms iterates again after adding the job sequence *gamma* to the forbidden in set Γ. Indeed, an objective function value greater than zero means that the current solution presents some differences between the arrival times of the same product components. If the feasible solution has a value of 0 (step 11), then a feasible solution for the 2-HSFA has been found because the current solution has all the product components arriving simultaneously at the assembly stage. Thus, the ${\bar{C}}_{\text{max}}$ is reduced by a percentage *β*′ to explore a tighter makespan. If no feasible solution is found, then the ${\bar{C}}_{\text{max}}$ is increased (step 14). The algorithm iterates until a maximum number of iterations without improvement is reached or a time limit is reached (verified in the inner while loop).

The assignment stage of the pull-matheuristic determines the job assignment to the machines at the different stages by minimizing the completion time of the jobs at each stage. In this parametric model, we do not consider the job sequence but only ensure that the proposed assignment is not forbidden and, thus, is not in the Γ set. For this, we solve the following parametric MILP named AS(Γ) where the variable $C_{km}^{l}$ corresponds to the maximum accumulated processing times in stage *k* ∈ *K*^*l*^, calculated considering all batches of component *l* ∈ *L* assigned to any machine.

$$\begin{array}{ccc} & \text{min}\sum_{l\in L}\sum_{k\in K^{l}}\sum_{m\in M_{k}^{l}}C_{km}^{l} & \end{array}$$ (13)

$$\begin{array}{ccc} & \sum_{j\in J^{l}}\sum_{m\in M_{k}^{l}}p_{jkm}^{l}y_{jkm}^{l}\leq C_{km}^{l} & k\in K^{l},l\in L \end{array}$$ (14)

(3)–(4)

$$\begin{array}{cc} \sum_{l\in L}\sum_{j\in J^{l}}\sum_{k\in K^{l}}\sum_{m\in M_{k}^{l}}y_{jkm}^{l}\leq \left|\gamma \right|-1 & \gamma \in \Gamma \end{array}$$ (15)

$$\begin{array}{ccc} & y_{jkm}^{l}\in \left\{0,1\right\} & j\in J^{l},m\in M_{k}^{l},k\in K^{l},l\in L\\ & C_{mk}^{l}\geq 0 & m\in M_{k}^{l},k\in K^{l},l\in L \end{array}$$

In the AS(Γ) mathematical model, the objective function (13) goal is to bound the processing time used in each stage by minimizing the sum of the maximum processing times for every component and every stage. Constraints set (14) establish the value of the $C_{mk}^{l}$ variables; these constraints do not consider the setup times because this model does not perform a schedule. Constraints set (15) make the connection between the AS(Γ) and the scheduling models in the matheuristic by forbidding all the unfeasible job sequences *γ* ∈ Γ. The last constraint sets establish the nature of the variables.

The scheduling stage pull-matheuristic is also a parametric MILP that determines the schedule of jobs for each machine at each stage of the 2-HFSA. For this, we consider the assignment solution $\bar{(}Y)=\left\{y_{jkm}^{l}\right\}$ yielded by the AS(Γ) model and the actual bound on the makespan ${\bar{C}}_{\text{max}}$. This model is named $\text{SS}(\bar{Y},{\bar{C}}_{\text{max}})$ and requires excess variables *D*(*i*, *j*) that represent the difference in the completion time of a pair of jobs forming a product at the assembly stage, (*i*, *j*) ∈ *N*.

$$\begin{array}{cc} & \text{min}\sum_{l\in L}\sum_{(i,j)\in N}D_{\left(i,j\right)} \end{array}$$ (16)

$$\begin{array}{ccc} & C_{ja}^{l}\leq {\bar{C}}_{\text{max}} & j\in J^{l},l\in L \end{array}$$ (17)

(5)–(9), (11)

$$\begin{array}{cc} x_{(i,j)km}^{l}\leq {\bar{y}}_{jkm}^{l} & i,j\in J^{l},m\in M_{k}^{l},k\in K^{l},l\in L \end{array}$$ (18)

$$\begin{array}{c} C_{jk}^{l}\geq S_{(i,j)km}^{l}+p_{jkm}^{l}{\bar{y}}_{jkm}^{l}+\tau_{(i,j)km}^{l}x_{(i,j)km}^{l}-B\left(1-x_{(i,j)km}^{l}\right)\\ i,j\in J^{l},k\in K^{l},m\in M_{k}^{l},l\in L \end{array}$$ (19)

$$\begin{array}{ccc} & C_{ia}^{1}-C_{ja}^{2}\leq D_{\left(i,j\right)} & \;(i,j)\in N \end{array}$$ (20)

$$\begin{array}{ccc} & C_{ja}^{2}-C_{ia}^{1}\leq D_{\left(i,j\right)} & (i,j)\in N \end{array}$$ (21)

$$\begin{array}{ccc} & x_{(i,j)km}^{l}\in \left\{0,1\right\} & i,j\in J^{l},m\in M_{k}^{l},k\in K^{l},l\in L \end{array}$$ (22)

$$\begin{array}{ccc} & C_{jk}^{l},S_{(i,j)km}^{l}\geq & i,j\in J^{l},m\in M_{k}^{l},k\in K^{l},l\in L \end{array}$$ (23)

$$\begin{array}{ccc} & D_{\left(i,j\right)}\geq 0 & (i,j)\in N \end{array}$$ (24)

In the $SS(\bar{Y},{\bar{C}}_{\text{max}})$ model, the objective function (16) minimizes the sum of the differences between the completion times of the pair of jobs forming a product at the assembly stage. The completion time of the jobs at the assembly stage is bounded by parameter ${\bar{C}}_{max}$ in the constraint set (17). Constraint sets (5)–(9) amd (11) are the ones presented previously, defining the job sequence in the stages of the 2-HFSA problem. Constraints (18) define the sequence variables with the parameter values ${\bar{y}}_{jkm}^{l}$ obtained by solving the AS(Γ) model. Constraints (19) determine the starting and completion times of the jobs in the different stages, taking into account the already computed jog assignment $\bar{Y}$. Eq sets (20) and (21) define the time difference *D*_(*i*, *j*)_ between each pair of jobs forming a product in the assembly stage. The rest of the equations are the variable’s nature.

Experimental results of the pull-matheuristic will be presented in Section 3.

### 2.3 GRASP for the 2-HFSA problem

The greedy randomized adaptive search procedure (GRASP) is a metaheuristic consisting of constructive iterations of greedily built randomized solutions that are then improved by a local search stage. GRASP was introduced in, and there are several surveys about this topic [39, 40].

Algorithm 2 displays our GRASP procedure to solve the 2-HFSA problem. While a maximum number of iterations is not reached, our GRASP generates a partial solution for HFS^2^ by adding jobs to the problem’s solution from a list of elements ranked by a greedy function according to the partial makespan per stage (GreedySolutionHFS^2^). Then, with GreedySolutionHFS^1^, we sequence the jobs in HFS^1^. As mentioned in the introduction, it is usual that one line is faster than the other. In our case study, HFS^1^ is the fastest, so we start the greedy solution with the HFS^2^ and then set the other line accordingly. Combining the two greedy functions forms a feasible solution for the 2-HFSA that is then improved by a local search procedure.

**Algorithm 2** GRASP Algorithm for 2-HFSA

1: Y*, X* ← ∅, $C_{\text{max}}^{*}\leftarrow \infty$

2: **for** *i* ≤ *MaxIter* **do**

3:  $\bar{Y},\bar{X}\leftarrow$ GreedySolutionHFS^2^

4: $\bar{Y},\bar{X}\leftarrow$ GreedySolutionHFS^1^ $\left(\bar{Y},\bar{X}\right)$

5:  *C*_max_← $\text{LocalSearch}(\bar{Y},\bar{X})$

6:  **if** $C_{\text{max}}^{*}>C_{\text{max}}$ **then** $Y^{*}=\bar{Y},X^{*}=\bar{X}$, $C_{\text{max}}^{*}=C_{\text{max}}$

7: **end for**

8: return $Y^{*},X^{*},C_{\text{max}}^{*}$

Algorithm 3 details the GreedySolutionHFS^2^ procedure that corresponds to the greedy construction of a partial solution by scheduling jobs to line HFS^2^. First, the candidate list *C* comprises the jobs in *J*^2^, and an empty solution is set. While the list is not empty, for each stage, each job *j* is greedily assigned to the best machine by evaluating its partial completion time in this stage (step 5). Then, we denote by $C_{\text{max}}^{J^{2}\backslash C\cup j}$ the partial makespan of the elements in *J*^2^\*C* union job *j*. To obtain variability in the candidate set of greedy solutions, the ranked jobs according to their corresponding $C_{\text{max}}^{J^{2}\backslash C\cup j}$ are placed in the restricted candidate list RCL (step 9), where *α* ∈ (0, 1) is responsible for the size of the RCL. A job j* is chosen randomly when building the solution (step 10). The completion time of the assembly stage for the HFS^1^ is then equal to the one obtained in the HFS^2^.

**Algorithm 3** GreedySolutionHFS^2^

1: Candidate list *C* = *J*^2^, current solution $\bar{Y}=\emptyset$ and $\bar{X}=\emptyset$

2: **while** *C* ≠ ∅ **do**

3:  **for** *j* ∈ *C* **do**

4:   **for** *k* ∈ *K*^2^ **do**

5:    Determine compatible machine $m\in M_{k}^{2}$ minimizing $C_{jk}^{2}$

6:   **end for**

7:   Compute partial makespan of HFS^2^: $C_{\text{max}}^{J^{2}\backslash C\cup j}$

8:  **end for**

9:  *RCL* composed by *j* ∈ *C* such that $C_{\text{max}}^{J^{2}\backslash C\cup j}<{\text{min}}_{j^{\prime }\in C}\{C_{\text{max}}^{J^{2}\backslash C\cup j\prime }\}+\alpha \left({\text{max}}_{j^{\prime }\in C}\left\{C_{\text{max}}^{J^{2}\backslash C\cup j\prime }\right\}-{\text{min}}_{j^{\prime }\in C}\left\{C_{\text{max}}^{J^{2}\backslash C\cup j\prime }\right\}\right)$

10:  Randomly select j* in the *RCL*

11:  Update $\bar{Y},\bar{X}$ with j* sequenced in the chosen machines of the stages

12:  $C_{j^{*}a}^{2}=C_{ia}^{1}$, for (*i*, j*)∈*N*

13:  *C* = *C*\*j*

14: **end while**

15: Return $\bar{Y},\bar{X}$

Production line one must end simultaneously with production line two; therefore, the constructive algorithm GreedySolutionHFS^1^ $\left(\bar{Y},\bar{X}\right)$ (pseudocode in Algorithm 4) takes the partial solution $\left(\bar{Y},\bar{X}\right)$ together with its completion time as input. The greedy scheduling construction of HSF^1^ is performed from the assembly stage to the first stage, assigning jobs to machines following the sequence order of HFS^2^ obtained by Algorithm 3. Algorithm 4 assigns each batch to a machine, using the best greedy insertion value in every stage. Note that this procedure may lead to completion time unfeasibility, which is solved by adjusting (increasing) the completion times in line HFS^2^.

**Algorithm 4** GreedySolutionHFS^1^ $\left(\bar{Y},\bar{X}\right)$

1: **for** *i* ∈ *J*^2^ ordered with respect to $\bar{(}X)$ **do**

2:  **for** *k* = *a*, …, 1 **do**

3:   Determine compatible machine $m\in M_{k}^{1}$ minimizing $C_{jk}^{1}$

4:   Update $\bar{Y},\bar{X}$ with the assignment of *i*

5:   **if** resulting partial solution is unfeasible **then**

6:    adjust completion times of (*i*, *j*) in HSF^2^

7:   **end if**

8:  **end for**

9: **end for**

10: Return $\bar{Y},\bar{X}$

The obtained solution after the two greedy construction phases is given as input to the Local Search (see Algorithm 2) as an attempt to reduce its objective function. In our GRASP, the local search takes the sequence of the jobs and their scheduling from HFS^2^ since it is the bottleneck line. Then, we apply the remove/insert operator for every job: we remove the job from its sequence and insert it at the end of the sequence in both lines HFS^1^ and HFS^2^ in its best compatible machine. We make their completion time coincide at stage *a* and then shift all the other jobs and update the total completion time. After obtaining all the possible neighbors of the current solution, we keep the best one and continue with the iterations of the GRASP algorithm.

**Fig 2:**
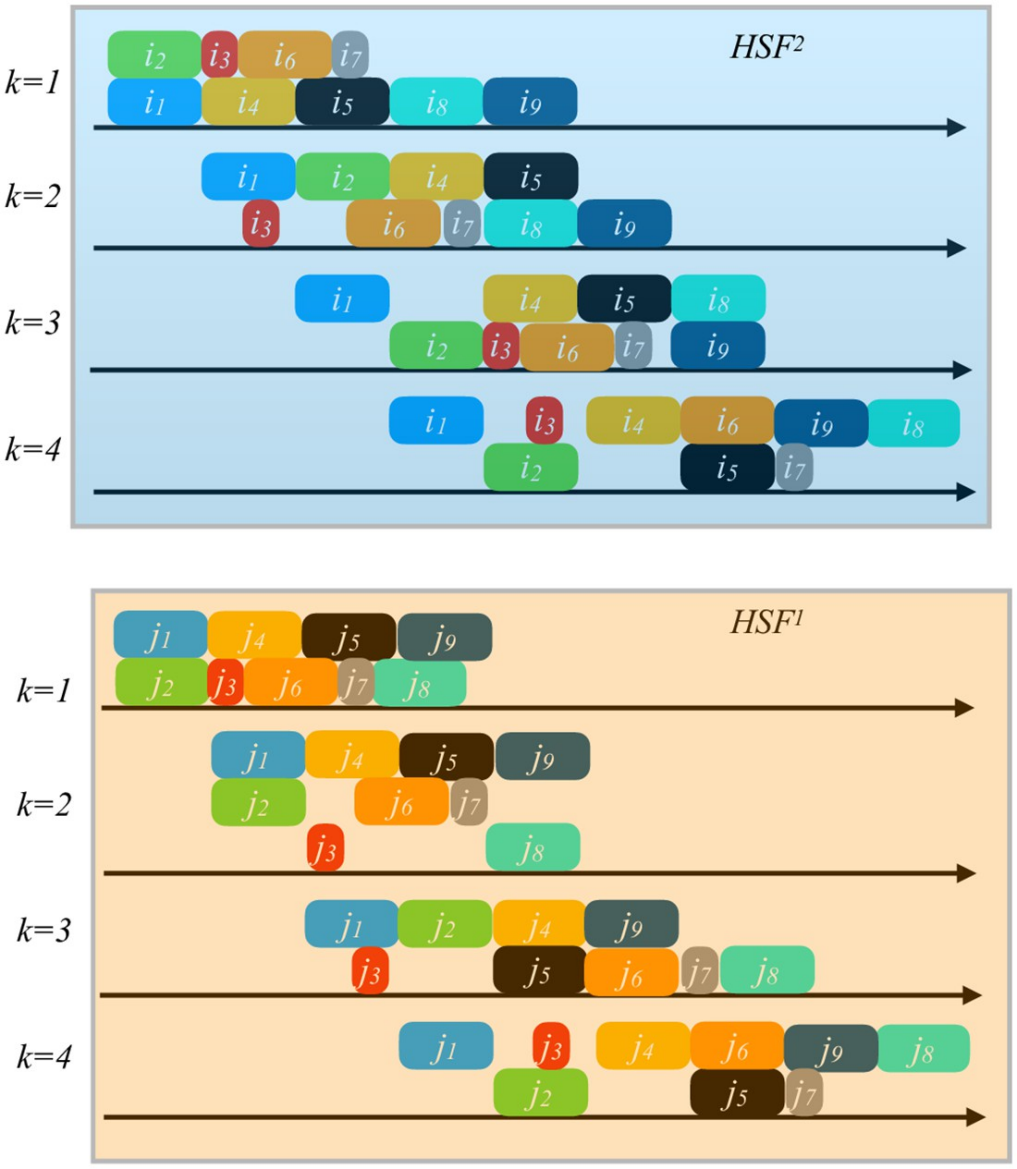
Solution of the 2-HFSA problem after applying our GRASP procedure to an instance with nine pairs of jobs, four stages in each HFS, and unrelated parallel machines.

Fig 2 represents a solution to the 2-HFSA problem after applying our GRASP procedure. The instance has nine pairs of jobs, four stages in each HFS, and unrelated parallel machines. Note that the completion times of each pair at the end of the last stage *k* = 4 of each HFS are simultaneous. This way, the pair of jobs can go into the assembly stage.

### 2.4 BRKGA for the 2-HFSA problem

Genetic Algorithms have been used to solve scheduling problems [41–43]. [44] present the Biased Random Key Genetic Algorithm, BRKGA. In this section, we present a BRKGA to solve the 2-HFSA problem.

As mentioned by [45, 46], BRKGAs start with a population of *p* chromosomes that will evolve with the next generations until *MaxG*. Each chromosome has *R* random keys for several generations until a total number of generations is reached. In a given generation *r*, the evolutionary process is as follows.

1. Each chromosome is decoded, and its objective value (or fitness) is computed.
2. The population is partitioned into an elite set containing *p*_*e*_ individuals with the best fitness values and the non-elite set with the rest of the individuals.
3. The population at generation *g* + 1 consists of *p*_*e*_ elite individuals of the current generation, *p*_*m*_ mutants that are randomly generated chromosomes, and *p*_*o*_ = *p* − *p*_*m*_ − *p*_*e*_ offspring chromosomes produced by crossover operation between random individuals with replacement, one from the elite and another from the non-elite set. In the crossover, the *v*-th allele of offspring *u* is defined by a uniform random number in [0, 1); if this value is larger than the biased probability *ρ* > 0.5, then offspring *u* inherits the *v*-th allele of its elite parent; otherwise, it inherits the *v*-th allele of the non-elite parent.

The encoding and decoding algorithms are the only problem-dependent stages of the BRKGA. For the 2-HFSA problem, each chromosome of the population will be a vector of size *R* = |*N*|(1 + |*K*^2^| + |*K*^1^|): the first |*N*| alleles represent the sequence of the jobs in both lines, the second |*K*^2^||*N*| alleles indicate the job assignment to the machines of each stage of HFS^2^, and the final |*K*^1^||*N*| alleles represents the job machine assignment for each batch for HFS^1^.

**Fig 3:**
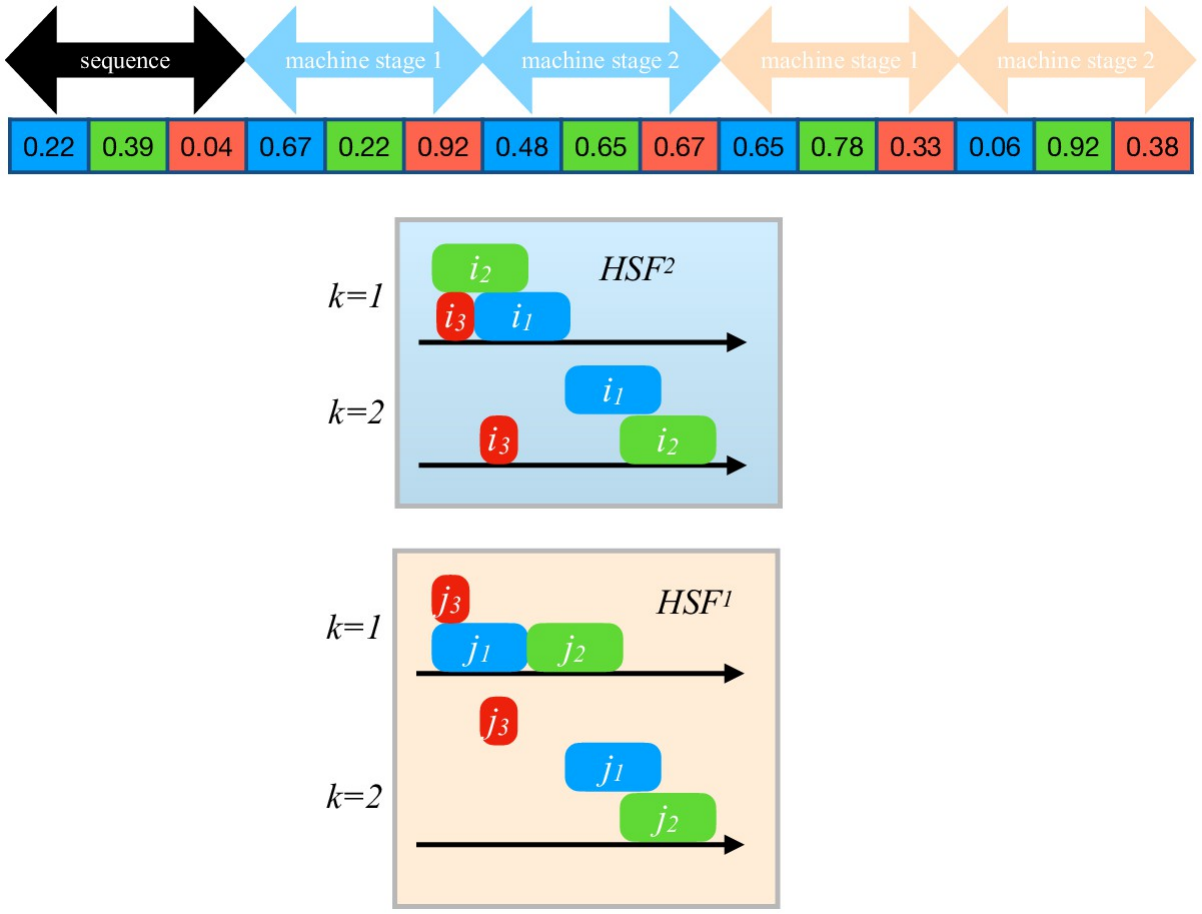
Random key decoding example for an instance with three pairs of jobs and two stages per line.

We use the decoding method to transform the [0,1) random key of size *R* and obtain a solution related to the 2-HFSA problem with its total completion time. Fig 3 shows an example of our decoding algorithm for an instance with three pairs of jobs and two stages per line. Note that HSF^1^ has three machines in stage 2. To obtain the sequence of the jobs in HFS^2^, we sort the first |*N*| alleles in non-decreasing order. In our example, the sequence would be (3,1,2). Then, the following |*k*^2^*N*| alleles indicate the machine to which the job will be assigned in the HFS^2^: the [0,1) interval is divided into the number of machines in the stage. Each machine is then associated with one of the subdivided intervals. In our example, stage 1 has two machines; thus, job *i*_2_ is in Machine 1 and *i*_3_ and *i*_1_ in Machine 2. Similarly, the final |*k*^2^*N*| alleles are for the HFS^1^. Note that for Stage 2, there are three machines. Once the jobs are sequenced and forced to end simultaneously at the end of the last stage of the lines, the makespan of the individual can be computed.

## 3 Results and discussion

This section describes the computational experiments to validate and compare the mathematical model with the proposed heuristic algorithms. First, we describe the instance’s characteristics. Then, we present a parameter-tuning procedure for the different algorithms. Finally, we compare the algorithm’s performance.

We used an Intel (R) Core^™^ processor with 2.9GHz and 8GB RAM speed for computational experimentation. The codes were implemented in C++, using Microsoft Visual Studio 2012 and ILOG CPLEX 12.5 as a solver for the mathematical model. All the instances and the detailed results are in https://doi.org/10.6084/m9.figshare.24246547.

Experimentation includes four types of instances. Each instance type’s size depends on the number of jobs, stages, and machines per stage. We create five instances of each type for a total of twenty instances. In Table 1, we present the values used for each type of instance: number of jobs |*N*|, number of stages for HSF^1^ and HFS^2^ (|*K*^1^| and |*K*^2^|, respectively), maximum number of machines per line ($\text{max}|M_{k}^{1}|$ and $\text{max}|M_{k}^{2}|$), processing times for HSF^1^ and HFS^2^, and setup times values in the last column. In each instance, the last stage is considered as the assembly stage. Type 3 instances emulate the usual production line of our case study, while Type 4 instances are rare but may sometimes occur. Since no similar article exists in the literature, comparing our approach with other approaches is difficult without removing strong components of our problem, like the assembly stage or the two related HFS. Nevertheless, we compare exact and approximated methods to evaluate our methodologies’ performance in terms of quality and time.

**Table 1:** Description of the instance benchmark.

Type	|*N*|	|*K*^1^|	|*K*^2^|	$\text{max}|M_{k}^{1}|$	$\text{max}|M_{k}^{2}|$	$p_{ikm}^{1}$	$p_{jkm}^{2}$	$\tau_{(i,j)km}^{l}$
1	[10,12]	2	2	4	3	[40, 70]	[10, 12]	[25, 45]
2	[29, 35]	3	3	5	5	[40, 70]	[15, 25]	[25, 45]
3	[50, 60]	5	6	10	12	[60, 90]	[20, 30]	[25, 45]
4	[90, 125]	12	14	25	29	[40, 65]	[10, 12]	[25, 45]

The algorithm parameters for our GRASP and the BRKGA were tuned using Calibra [47], a software based on a Taguchi Fractional design of experiments enhanced with a local search procedure. Table 2 shows the test values and the results obtained by Calibra for our algorithms. For each parameter, the *Range* is the interval tested by Calibra, and column *Value* is the yielded parameter. For the GRASP, only two parameters must be tuned: the size of the restricted candidate list controlled by *α* and the maximum number of iterations.

**Table 2:** Parameter tuning for the GRASP and the BRKGA algorithms obtained by Calibra.

GRASP			BRKGA		
Parameter	Range	Value	Parameter	Range	Value
*α*	[0,1)	0.3	*p*	[100,1000]	887
*MaxIter*	[100, 1000]	763	*p*_*m*_	[0, 0.3*p*⌉	⌈0.3*p*⌉
			*p*_*e*_	⌈0, 0.7*p*⌉	⌈0.6*p*⌉
			*ρ*	[0.5,1]	0.9
			*MaxG*	[10, 100]	78

For the pull-matheuristic, at each iteration *β*, *β*′ are uniformly drawn ∈ [0, 0.5), the maximum number of iterations without improvements is set to five, and a total of three hours time limit of the inner loop.

Table 3 presents the results obtained with the mathematical model and the three approximated solution approaches that we proposed. The first column corresponds to the instance type. Columns 2 to 4 show the results obtained with the mathematical model. Columns 5 to 9 are for the pull-matheuristic, the next four columns are for the GRASP, and the last four are for the BRKGA. For the mathematical model, we present the objective value (“Obj.”), the percentage gap (“Gap”) computed as 100(best dual—best feasible solution)/(best feasible solution), and the time in seconds (“T.”). We executed the algorithm 20 times for the three approximated algorithms because of their random components. We present the best objective value (columns“Obj.”), the average objective function values (columns “Ave.”), the average time in seconds (“T.”), and the percentage difference (“Dif.”) with the best feasible solution found by all the methods computed as 100(Obj.—best Obj.)/(best Obj.). The best objective function values are marked in bold.

**Table 3:** Experimental results obtained with the mathematical model, the pull-matheuristic, the GRASP, and the BRKGA.

Type	Mathematical model			Pull-matheuristic				GRASP				BRKGA			
	Obj.	T.	Gap	Obj.	Ave.	T.	Dif.	Obj.	Ave.	T.	Dif.	Obj.	Ave.	T.	Dif.
1	**1365.8**	3600	68	1389.1	1396.3	2117	1	1420.3	1420.3	0	2	1402.9	1411.6	0	1
	891.6	3600	24	**880.9**	885.4	1176	0	949.81	1033.2	0	8	881.6	929.1	1	0
	1297.9	3600	73	**1289.8**	1300.9	2287	0	1320.3	1320.3	0	2	1300.2	1308.2	1	1
	1106.7	3600	68	**1132.6**	1137.2	2227	0	1200.5	1200.5	0	6	1154.3	1165.1	0	2
	1194.0	3600	70	**1197.2**	1213.2	2241	0	1229.0	1236.1	0	3	1212.0	1218.5	0	1
2	-	3600	-	6589.0	6698.5	8145	38	**4775.2**	4826.7	1	0	4778.1	4799.2	1	0
	3411.0	3600	97	4693.7	5017.5	7645	71	2779.0	2829.5	1	1	**2749.8**	2924.0	8	0
	4722.1	3600	100	5131.7	5205.0	7294	26	**4061.0**	4067.9	1	0	4062.1	4067.1	1	0
	4180.9	3600	100	5368.2	5368.2	7585	46	3674.5	3700.3	2	0	**3665.9**	3678.7	4	0
	-	3600	-	6388.9	6408.2	8121	32	4824.7	4863.1	1	0	**4824.2**	4835.2	2	0
3	-	3600	-	3754.3	3754.3	7958	5	3564.1	3667.8	14	0	3638.2	3917.2	24	2
	-	3600	-	5310.2	5310.2	13225	41	**3755.3**	4199.9	19	0	3805.7	4228.3	28	1
	-	3600	-	3590.6	3590.6	9243	7	**3363.4**	3857.1	14	0	3457.5	3964.6	23	3
	-	3600	-	5271.0	5271.0	7117	76	**2990.0**	3229.9	11	0	3047.8	3309.1	22	2
	-	3600	-	4037.5	4037.5	7659	1	**3983.1**	4199.8	15	0	3985.1	4127.7	24	0
4	-	3600	-	33730.5	33730.5	3490	161	**12900.4**	14201.2	291	0	14241.4	18393.7	102	10
	-	3600	-	-	-	40300	-	**11085.0**	12486.8	320	0	14103.2	20660.2	107	27
	-	3600	-	-	-	45242	-	**12893.4**	14377.9	338	0	16528.4	25785.5	110	28
	-	3600	-	-	-	-	-	**14888.0**	16095.6	493	0	22467.8	45194.9	134	51
	-	3600	-	-	-	-	-	**11638.5**	12854.1	360	0	14443.6	19757.2	113	24
**Average**	**75**			**32**				**1**				**8**			

For small instances of Type 1, the mathematical model solved with the integer linear solver obtains solutions with a gap average of less than 61%. The solver could not find feasible solutions for many Type 2 instances, moreover, the average gaps are almost 100%. It is important to highlight that the relevance of the model lies in guaranteeing the optimal solution, even if it is only for smaller instances.

The pull-matheuristic finds high-quality solutions for Type 1 instances and better solutions than the exact mathematical model, considering that the gaps were large. Note that the pull-matheuristic resides on exact models; therefore, as expected, it presents more computational time than the GRASP and the BRKGA ones. The pull-matheuristic yields feasible solutions for Type 2 and 3 ones. However, it could only solve one for Type 4 instances within the time limit. The complexity of the instances for the pull-matheursitic resides mainly in the number of jobs in the flow shops.

Regarding the GRASP and the BRKGA, both algorithms are very efficient in terms of time compared with the mathematical model and the pull-matheuristic. The GRASP algorithm finds the best solutions in the Type 3 and 4 instances, while the BRKGA performs well for the Type 2 instances. Nevertheless, the computational time of the BRKGA is very efficient, even compared to the GRASP for the larger instances. When solved with GRASP or the BRKGA, the complexity of an instance is in the number of jobs that must be processed and assembled in the system. Nevertheless, note that the BRKGA yields solutions in less than two minutes for real-life applications.

## 4 Conclusions

This article studies the 2-Hybrid Flow Shop with Assemble stage, where two HFS must be coordinated to assemble batches of terminated products at the end of the lines. Each product comprises two jobs, each produced in one of the HFSs. In both HFSs, the jobs will be processed in multiple stages with multiple parallel machines to minimize the scheduling time span. The jobs forming a product must arrive at the assembly line simultaneously.

To the best of our knowledge, there is no similar problem in the literature, and since it is based on a real case study, we aimed to solve it exactly and heuristically. This paper proposes a mathematical model for the 2-HFSA and three approximated solution approaches: a matheuristic based on the decomposition of the exact mathematical model and two metaheuristic algorithms, a GRASP and a BRKGA.

The computational experiments include up to 14 stages and 29 machine instances, including a stage of assembly. According to a literature review, no studies include experimentation with the size of Type 4 instances, which emulates some of the real case scenarios. The computational results give evidence of the suitability of that GRASP-based methodology for company implementation due to the computation elapsed time required by the algorithm for quality solutions.
